# Lung ultrasound: a useful tool in diagnosis and management of bronchiolitis

**DOI:** 10.1186/s12887-015-0380-1

**Published:** 2015-05-21

**Authors:** Vincenzo Basile, Antonio Di Mauro, Egisto Scalini, Paolo Comes, Ignazio Lofù, Michael Mostert, Silvio Tafuri, Mariano M. Manzionna

**Affiliations:** Pediatric Unit, Maternal and Child Health Department, “S. Giacomo” Hospital, ASL BA, Largo Simone Veneziani, 21, Monopoli (Bari), Italy; Neonatology and Neonatal Intensive Care Unit, Department of Biomedical Science and Human Oncology, University of Bari “Aldo Moro”, Bari, Italy; Radiodiagnostic Unit, “S. Giacomo” Hospital, ASL BA, Largo Simone Veneziani, 21, Monopoli (Bari), Italy; Pediatric Unit University of Turin, Turin, Italy; Section of Hygiene, Department of Biomedical Sciences and Human Oncology, University of Bari “Aldo Moro”, Bari, Italy

**Keywords:** Chest ultrasound, Sonographic interstitial syndrome, Bronchiolitis

## Abstract

**Background:**

Clinical assessment is the gold standard for diagnosis of bronchiolitis. To date, only one study found LUS (Lung Ultrasound) to be a valuable tool in the diagnosis of bronchiolitis. Aim of this study is to evaluate the accuracy of lung ultrasonography in the diagnosis and management of bronchiolitis in infants.

**Methods:**

This was an observational cohort study of infants admitted to our Pediatric Unit with suspected bronchiolitis. A physical examination and lung ultrasound scans were performed on each patient. Diagnosis and grading of bronchiolitis was assessed according to a clinical and a ultrasound score. An exploratory analysis was used to assess correspondence between the lung ultrasound findings and the clinical evaluation and to evaluate the inter-observer concordance between the two different sonographs.

**Results:**

One hundred six infants were studied (average age 71 days). According to our clinical score, 74 infants had mild bronchiolitis, 30 had moderate bronchiolitis and two had severe bronchiolitis. 25 infants composed the control group. Agreement between the clinical and sonographic diagnosis was good (90.6 %) with a statistically significant inter-observer ultrasound diagnosis concordance (89.6 %).

Lung ultrasound permits the identification of infants who are in need of supplementary oxygen with a specificity of 98.7 %, a sensitivity of 96.6 %, a positive predictive value of 96.6 % and a negative predictive value of 98.7 %. An aberrant ultrasound lung pattern in posterior chest area was collected in 86 % of infants with bronchiolitis. In all patients clinical improvement at discharge was associated with disappearance of the previous LUS findings. Subpleural lung consolidation of 1 cm or more in the posterior area scan and a quantitative classification of interstitial syndrome based on intercostal spaces involved bilaterally, good correlate with bronchiolitis severity and oxygen use.

**Conclusions:**

The lung ultrasound findings strictly correlate with the clinical evaluations in infants with bronchiolitis and permit the identification of infants who are in need of supplementary oxygen with high specificity. Scans of the posterior area are more indicative in ascertaining the severity of bronchiolitis.

**Trial registration:**

Clinical Trial Registration NCT01993797

## Background

Bronchiolitis is the most common lower respiratory tract illness that affects infants and children <2 years of age. It is usually caused by a viral infection with a peak of incidence and morbidity among infants of 1–3 months of age [1].

The diagnosis of bronchiolitis should be made on the basis of medical history and a clinical examination. Up to date, there are no serological or radiological signs included in the diagnostic work-up [2].

The pathophysiology of bronchiolitis is characterized by edema, an increased production of mucus and necrosis of the infected epithelial cells of the small airways that cause a heterogeneous obstruction of the distal bronchioles.

This process leads to a reduction in the air content in the lung with impaired diffusion across the blood-gas membrane and ventilation-perfusion inconsistency [3].

Lung ultrasound (LUS) is performed in adult and pediatric care for the evaluation of several cardiopulmonary conditions and with respect to the use of X-rays offers advantages as concerns the employment of ionizing radiation [4–7].

A healthy, normally-aerated lung reflects ultrasound beams due to its high acoustic impedance and shows only an anatomic indication of a hyper-reflective pleural line that slides, with breathing, towards the interface between tissue and air. A normal lung also reveals horizontal artifacts below the pleural line, called A lines, due to reverberations of ultrasound beam [8].

The gradual passage from a dry to a wet lung allows the reflection of the ultrasound beam and leads to the formation of vertical artifacts called B-lines [9]. An increasing concentration of vertical artifacts corresponds with the increasing extent of lung congestion and permits a quantification of pulmonary interstitial syndrome [10, 11].

The areas of lung dysventilation with an absence of alveolar air are visualized in the form of consolidations adjacent to the pleural line [12].

To date, only one study by Caiulo et al. found LUS to be a valuable tool in the diagnosis of bronchiolitis and at present LUS is not recommended for its management [13].

This study aims to compare the agreement between lung ultrasonography and clinical score in the diagnosis of infants with suspected bronchiolitis. In addition the present study explores the potential of ultrasound in bronchiolitis management in predict need for oxygen supplementation.

## Methods

This is an observational cohort study, performed at the Pediatric Unit of S. Giacomo Hospital of Monopoli (Bari, Italy) in association with the Radiology Department.

The local Ethics Committee and the Institutional Review Board of the S. Giacomo Hospital in Monopoli (Bari, Italy) approved the protocol. Written informed parental consent was obtained.

All patients were admitted from January 2010 to December 2013 with history, signs and symptoms of suspected bronchiolitis, according to the American Academy of Pediatrics [1, 2]. They all then underwent a routine clinical evaluation and a Rapid Test for Syncytial Respiratory Virus (SRV) (BinaxNOW®SRV). Infants admitted for routine hip and kidney echography composed the control group.

The attending physician assessed the overall clinical impression on the basis of a modified previously published protocol for bronchiolitis [14] (Table 1).

**Table 1:** Clinical score

Clinical score	0	1	2	3
Respiratory rate	<50	50 - 60	61-69	>70
Dyspnea	Normal feeding	Difficulty feeding	2 of the following: difficulty feeding, pallor, perioral cyanosis OR agitation.	2 of the following: cyanosis, stopped feeding OR drowsiness.
Use of accessory respiratory muscles	None	Subcostal or intercostal retractions	2 of the following: subcostal, intercostal, substernal retractions OR nasal flaring	3 of the following: subcostal, intercostal, substernal, suprasternal, supraclavicular retractions OR nasal flaring
Auscultation	Normal breathing	End-expiratory wheeze only OR crackles.	Expiratory wheeze and/or crackles	Inspiratory and expiratory wheeze OR diminished breath sounds OR both

Tachypnea, signs of dyspnea, use of accessory respiratory muscle and aberrant auscultation are thought to be the best clinical signs of bronchiolitis.

According to our clinical score, the diagnosis and grading of bronchiolitis was:

A. Mild bronchiolitis: score 1–4
B. Moderate bronchiolitis: score 5–8
C. Severe bronchiolitis: score 9–12
D. Healthy infant: score 0

To complete and widen the clinical evaluation, chest ultrasound scans were acquired by a pediatrician (VB) and a radiologist (PC) with a 10–12 MHZ linear transducer (LOGIQ P5 portable ultrasound system). All sonographers were equally skilled in chest scans and were unaware of the clinical score. The unavailability of a sonographer in the pediatric and radiology unit was considered exclusion criteria. We excluded patients with other major pathologies or previous chest-radiographs confirming pneumonia. In case of preterm intants, we excluded patients affected by bronchopulmonary dysplasia whose ultrasound pattern could be similar to bronchiolitis ultrasound appearance.

Ultrasonography examinations were performed following the methodology previously described by Copetti, Cattarossi et al. [15–17]. Both longitudinal and transversal sections were collected on the anterior, lateral, and posterior chest wall.

**Fig. 1:**
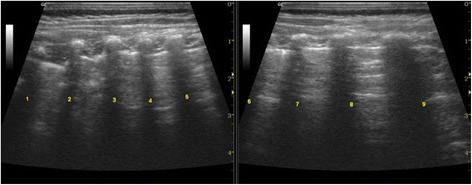
Quantitative classification of echographic interstitial syndrome

Data were classified according to the study protocol, on the basis of a proposed echographic score (Table 2). We decided to adopt this echographic score with the aim of answering a few simple queries about some bronchiolitis ultrasound findings that emerged from previous evaluations, such as a characteristic pattern of viral infections [18]; a presence of lung sliding with B-lines, confluent B-lines and sub pleural consolidations (Fig. 1) which were thought to be the best ultrasound signs of bronchiolitis [19].

**Table 2:** Bronchiolitis ultrasound score

US score		0	1	2
Anterolateral data		Normal lung sliding with horizontal artifacts (A-lines).	Diffuse and dishomogeneous interstitial syndrome with confluent, multiple B lines and spared areas.	Diffuse and dishomogeneous interstitial syndrome and/or subpleural lung consolidations.
		Vertical artifacts (B-lines) in limited number or absent.		
Paravertebral/ posterior data	Interstitial syndrome	Individual B line or absent	Focal, multiple B-lines	Confluent, multiple B lines
	Extension on interstitial syndrome	0-6 bilaterally involved intercostal spaces	6-12bilaterally involved intercostal spaces	>12 bilaterally involved intercostal spaces
	Presence of subpleural lung consolidation	Absent	Subcentimeter-subpleural lung consolidation	Subpleural lung consolidation of 1 cm or more

An accurate scanning of the posterior and paravertebral areas of the thorax was performed to increase the accuracy of the US examination [20].

**Fig. 2:**
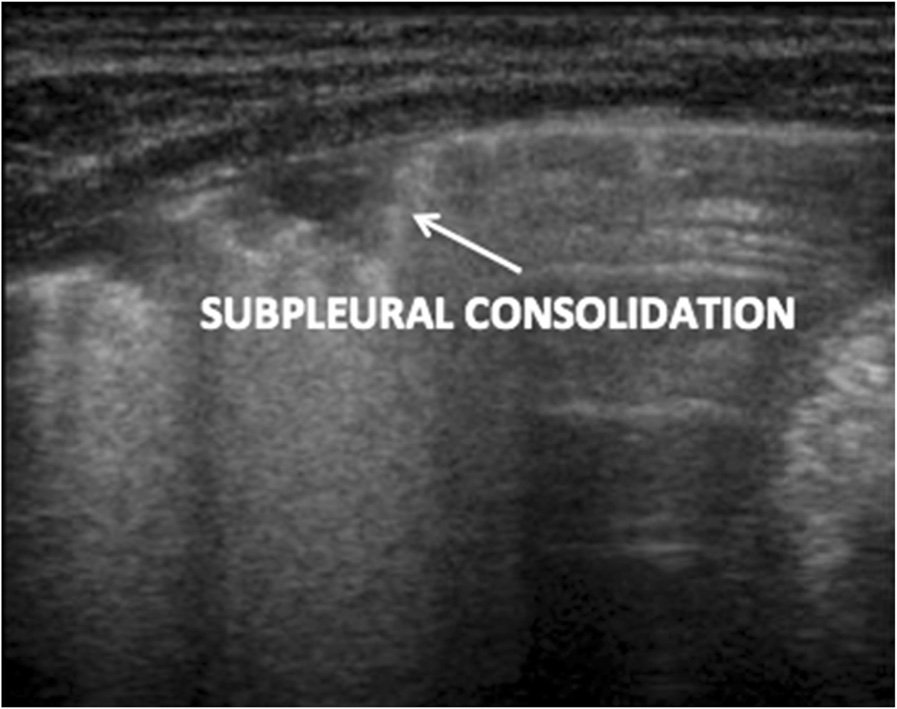
Subpleural consolidation

A quantitative classification of echographic interstitial syndrome was proposed, based on the extent of lung involvement, using the bilaterally involved posterior intercostal spaces as coordinates (Fig. 2).

According to the ultrasound findings, diagnosis and severity of bronchiolitis were assessed as follows:

A. Mild bronchiolitis: score 1–3
B. Moderate bronchiolitis: score 4–6
C. Severe bronchiolitis: score 7–8
D. Normal lung ultrasound pattern: score 0

Reference diagnosis made by sonographers, who independently reviewed the ultrasound findings, was collected and inter-observer concordance was assessed. Furthermore, the ultrasound score was correlated with the clinical data to estimate the agreement between clinical and echographic diagnosis.

The attending physician was unaware of the LUS score and was responsible for treatment decisions. Hypoxia due to ventilation/perfusion incongruity was evaluated by way of arterialized capillary blood gas determinations. Normal values for capillary blood oxygen tension were assumed to be >45 mmHg in air. Pulse oximetry (PM-7000, Masimo SET®) was used for monitoring trends in oxygenation. In accordance with local protocols, the infants were treated with oxygen supplementation when supplementary O_2_ was needed to keep saturation at >94 % (<90 if asleep) or capillary blood oxygen tension at <45 mmHg.

All patients underwent an echographical follow-up in order to obtain data on the disappearance of ultrasound pulmonary abnormalities during the course of the disease. At discharge, the US Score was recalculated. Clinical and ultrasonographical evaluation was performed even on infants without signs of bronchiolitis, admitted for routine hip and kidney echography (control group).

### Statistical analysis

Cohen’s kappa coefficient was calculated to assess agreement on diagnosis between the attending physician and the pediatric sonographer. The sonographer inter-observer concordance was also assessed.

According to recent literature^2^, 25 % of infants hospitalized for bronchiolitis need oxygen supplementation. With an α level of 0.05 and a power of 90 % a sample size of 19 infants was needed.

We calculated the specificity, sensitivity, positive predictive value and negative predictive value of the sonographic profile to predict the need for supplementary oxygen also when during acquiring the scan, saturation was more than 94 % or capillary blood oxygen tension was more than 45 mmHg. We defined true positive (TP) as US score >3 indicating that supplementary O_2_ was required. We defined true negative (TN) as US score <3 indicating that supplementary O_2_ was not required. False positive (FP) as US score >3 indicating that supplementary O_2_ was not required and false negative (FN) as US score <3 indicating that supplementary O_2_ was required.

The categorical data were expressed as counts and percentages. As regards the ultrasound findings, the *x*^2^ test was used to compare the percentages between different groups: mild bronchiolitis vs. moderate-severe bronchiolitis; infants in need of oxygen supplementation vs infants not in need of oxygen supplementation; SRV positive bronchiolitis vs SRV negative bronchiolitis.

For all tests, *P* <0.05 was considered significant.

## Results

One hundred eighteen infants were eligible for the study. Six were excluded for unavailability of sonographer, three for chest X-ray- diagnosed pneumonia, two for other concomitant pathologies.

One hundred six infants, aged from 9 to 239 days of life (median 71, mean: 87.4 ± 59.3) were enrolled.

Seventy three were positive for SRV. Seven infants were preterm newborns.

The characteristics of the infants at the time of enrollment are described in Table 3.

**Table 3:** Study population

Mode of delivery					
	Vaginal	64 (60 %)	Age		
	Cesarean	42 (40 %)		<3 month	40 (38 %)
Gender				>3 month	66 (62 %)
	Male	59 (56 %)	Use of drugs before enrollment		
	Female	47 (44 %)		Yes	46 (43 %)
				No	60 (57 %)
Feeding					
	Bottlefed	43 (41 %)	VRS		
	Breastfed	63 (59 %)		Yes	73 (69 %)
Gestational age				No	33 (31 %)
	Preterm	7 (7 %)	Auscultation of lung		
	Term	99 (93 %)		Normal	10 (9 %)
Family history of atopy				Wheeze	36 (34 %)
	Yes	25 (24 %)		Crackles	41 (39 %)
	No	81 (76 %)		Both	19 (18 %)

The control group was composed by 25 infants (11 males, 14 females). Ages ranged from 32 to 160 days of life. No one of the control group was positive for SRV. Only one was preterm newborns.

According to our clinical score performed by the attending physician: 76 infants had mild bronchiolitis, 27 had moderate bronchiolitis, and three had severe bronchiolitis.

According to our US score performed by the pediatric sonographer: 68 infants had mild bronchiolitis, 26 had moderate bronchiolitis, three had severe bronchiolitis and nine had a normal ultrasound pattern.

Agreement between the attending physician and the pediatric sonographer on the severity of bronchiolitis was high (agreement: 90.6 %; expected agreement: 52.3 %; K = 0.8; Std error = 0.0765; z = 10.19; *p* = 0.000).

According to our US score performed by the radiologist sonographer: 65 infants had mild bronchiolitis, 26 had moderate bronchiolitis, three had severe bronchiolitis and 12 had a normal pattern. Three of these 12 infants scanned by radiologist was considered with a mild bronchiolitis by pediatric sonographer.

Inter-observer concordance on the basis of the US findings between the two different sonographers was excellent (Cohen’s kappa coefficient: agreement = 89.6 %; expected agreement 46.4 %; K = 0.8; Std error = 0.07; z = 11.33; *p* = 0.000).

On the basis of our protocol on bronchiolitis, 29 (27 %) infants were in need of oxygen supplementation. LUS permits the identification of those infants that are in need of supplementary oxygen with a specificity of 98.7 % (95 % CI: 93 % to 99.8 %), a sensitivity of 96.6 % (95 % CI: 82.2 % to 99.4 %), a positive predictive value of 96.6 % (95 % CI: 82.2 % to 99.4 %) and a negative predictive value of 98.7 % (95 % CI: 92.95 % to 99.8 %).

In all patients with clinical sign of bronchiolitis, 56/106 (58 %) scans revealed alterations only in the posterior and paravertebral areas against 6/106 (6 %) only in the anterior areas. 35/106 (33 %) infants revealed alterations in both the posterior and anterior areas. 9/106 (8 %) scans revealed no alteration in either the posterior or anterior areas.

The distribution of LUS findings and scan position between the different groups are presented, expressed as counts and percentages, in Tables 4, 5 and 6.

**Table 4:** Quantitative classification of echographic interstitial syndrome between the different groups: clinically mild bronchiolitis vs. moderate-severe bronchiolitis; infants in need of oxygen supplementation vs infants in no need of oxygen supplementation

LUS data	Mild	Moderate	χ^2^ test	*p*	Oxygen	Oxygen	χ^2^ test	*P*
		Severe			No	Yes		
Less than 6 bilaterally involved intercostal spaces in the posterior and paravertebral area of the lung	61/76	3/30	44.3	0.00	61/77	3/29	41.7	0.00
	(80 %)	(10 %)			(79 %)	(10 %)		
6 to 12 bilaterally involved intercostal spaces in the posterior and paravertebral area of the lung	15/76	18/30	16.2		16/77	17/29	14	0.00
	(20 %)	(60 %)		0.00	(21 %)	(59 %)		
Up to 12 bilaterally involved intercostal spaces, in the posterior and paravertebral area of the lung	0/76	9/30	24.9	0.00	0/77	9/29	26.1	0.00
	(0 %)	(30 %)			(0 %)	(31 %)

**Table 5:** Subpleural lung consolidation in the posterior area of the lung between the different groups: clinically mild bronchiolitis vs. moderate-severe bronchiolitis; infants in need of oxygen supplementation vs infants in no need of oxygen supplementation

LUS data	Mild	Moderate	χ^2^ test	*p*	Oxygen	Oxygen	χ^2^ test	*P*
					No	Yes		
		Severe						
No presence of subpleural lung consolidations in the posterior and paravertebral area of the lung	53/76	5/30	24.4	0.00	53/77	5/29	22.6	0.00
	(70 %)	(17 %)			(69 %)	(18 %)		
Subcentimetersubpleural lung consolidations in the posterior and paravertebral area of the lung	20/76	12/30	1.9	0.16	20/77	12/29	2.3	0.1
	(26 %)	(40 %)			(26 %)	(41 %)		
Subpleural lung consolidation of 1 cm or more in the posterior and paravertebral area of the lung	3/76	13/30	26.4	0.00	4/77	12/29	21.5	0.00
	(4 %)	(43 %)			(5 %)	(41 %)

**Table 6:** LUS findings between SRV positive bronchiolitis vs SRV negative bronchiolitis

LUS data	SRV neg	SRV pos	*x* ^2^ test	*P*
Normal lung sliding with horizontal artifacts (A-lines), and vertical artifacts (B-lines) in limited number or absent in the anterolateral area of the lung	27/33	37/73	9.21	0.00
	(82 %)	(51 %)		
Diffused and dishomogeneous interstitial syndrome with confluent, multiple B lines and spared areas in the anterolateral area of the lung	5/33	30/73	6.92	0.00
	(15 %)	(41 %)		
Diffused and dishomogeneous interstitial syndrome and/or subpleural lung consolidations in the anterolateral area of the lung	1/33	6/73	0.9	0.31
	(3 %)	(8 %)		
Less than 6 bilaterally involved intercostal spaces in the posterior and paravertebral area of the lung	22/33	42/73	0.7	0.3
	(67 %)	(58 %)		
6 to 12 bilaterally involved intercostal spaces in the posterior and paravertebral area of the lung	9/33	24/73	0.3	0.5
	(27 %)	(33 %)		
Up to 12 bilaterally involved intercostal spaces, in the posterior and paravertebral area of the lung	2/33	7/73	0.3	0.5
	(6 %)	(9 %)		
No presence of subpleural lung consolidations in the posterior and paravertebral area of the lung	22/33	36/73	2.7	0.09
	(67 %)	(49 %)		
Subcentimetersubpleural lung consolidations in the posterior and paravertebral area of the lung	7/33	25/73	1.8	0.17
	(21 %)	(34 %)		
Subpleural lung consolidation of 1 cm or more in the posterior and paravertebral area of the lung	4/33	12/73	0.3	0.5
	(12 %)	(17 %)		
No interstitial syndrome ultrasound signs in the posterior and paravertebral area of the lung	8/33	14/73	0.3	0.5
	(24 %)	(19 %)		
Focal, multiple B-lines in the posterior and paravertebral area of the lung	20/33	37/73	0.9	0.3
	(61 %)	(51 %)		
Confluent, multiple B lines in the posterior and paravertebral area of the lung	5/33	22/73	2.6	0.1
	(15 %)	(30 %)		
Only posterior area	23/33	33/73	5.4	0.01
	(70 %)	(45 %)		
Only anterior area	3/33	3/73	1.06	0.3
	(9 %)	(4 %)		
Both anterior and posterior area	3/33	32/72	12.7	0.00
	(9 %)	(44 %)		
No alteration	4/33	5/72	0.8	0.3
	(12 %)	(7 %)		

In all patients (100 %) the clinical improvement at discharge was associated with the disappearance of the previous LUS findings and a lower US score.

Ultrasound findings in the control group were all compatible as a normal pattern: subpleural lung consolidations or compact B-lines were observed in 0/25 (0 %) infants, an Individual B line without any pathological significance was observed in 5/25 (20 %) infants.

## Discussion

Traditionally, the evaluation of the chest is considered possible only through exposure to radiation. Not long ago, the text “*Harrison’s principles of internal medicine*” considered the lung unsuitable for ultrasonographic examination [21]. Nevertheless, lung ultrasound has recently been applied to the diagnosis and management of several diseases and many authors have conducted several studies with promising results, all analyzed in the recent “*International evidence-based recommendations for point-of-care lung ultrasound*” [22].

We demonstrated that sonographers were able to accurately identify bronchiolitis using LUS with a good agreement on its severity between clinical and ultrasound evaluations. Our study confirms LUS as a valuable tool in the diagnosis and management of bronchiolitis and upholds previous conclusions made by Caiullo et al. that recently showed how the use of ultrasound can drastically reduce the need for chest X-ray [10].

Differently from Caiulo et al. [11] who reported data from a single operator, we used different ultrasound operators to evaluate diagnostic accuracy and reproducibility. It is important that every ultrasound examination is carried out in a standard manner and that the same anatomical or artifactual signs can be collected from one patient to another. We identified an echographic score with the aim of elaborating a unified and reproducible approach to LUS by different sonographers which permits consistency when different physicians scan one patient. Our calculated Cohen’s Kappa was 0.8, which means that the inter-observer agreement between the pediatric operator’s interpretation and that of the blinded radiologist operator was excellent. The evidence of an excellent inter-observer agreement between two operators suggests that lung ultrasound is not only easy to perform but also easy to read. Pediatric and radiologist diagnosis differs only in 3/12 cases where a normal US pattern according to radiologist was considered a mild bronchiolitis US pattern by pediatric sonographer. All these 12 infants did not need oxygen supplementation in the next hours, despite clinically diagnosed as affected by mild bronchiolitis and hospitalized. We speculate that pediatric sonographer over-diagnosed US data, influenced by clinical signs because could not avoid observing the patients. Based on our data, clinicians were able to detect infants that will be in need of supplementary oxygen using ultrasonography with high specificity and sensitivity. It means that we can claim a possible prognostic use of LUS in discriminate patients in need of hospitalization and oxygen supplementation, when saturation is more than 94 % and capillary blood oxygen tension was >45 mmHg in air. The use of LUS as a rapid and efficient screening tool to grant respiratory assistance in a timely fashion was already demonstrated in neonatal respiratory distress by Raimondi et al. [23].

A great percentage of our infants with bronchiolitis presented ultrasound anomalies on posterior and paravertebral scans. This data can be explained because the posterior area is more involved in bronchiolitis pathophysiology due to the gravity of obligate supine newborns and infants. Up to date, to our knowledge, it is the first time that the ultrasound involvement of paravertebral areas is described.

Our quantitative classification of echographic interstitial syndrome (Fig. 1) based on posterior bilaterally involved intercostal spaces correlates well with the need for oxygen supplementation and the severity of the bronchiolitis (Table 4).

Particular importance has to be given to infants with a subpleural lung consolidation of 1 cm or more in the posterior area scan (Fig. 2) as a great percentage of these are in need of oxygen supplementation (Table 5).

Our LUS data showed a great percentage of both posterior and anterior areas involved in patients positive for SRV and a great percentage of no alteration in LUS data in patients negative for SRV. Nevertheless, we couldn’t find any particular US findings related to SRV infections and, according to previous data published by Tsung et al. [18], it is unknown if different viruses have specific LUS patterns (Table 6).

We acknowledge some limitations of our study. Firstly, we had only three infants diagnosed with severe bronchiolitis. Secondly, our study population age span is considerably wide and is mainly represented by term infants and not by pre-terms who are at a higher risk of developing more serious illnesses. Further research into an intensive care setting and into a wider population are required in order to improve our findings. Thirdly, in this study, experienced physicians in LUS performed the ultrasound evaluations, and the results might not be extrapolated to all pediatricians. However, other authors have reported that physicians without previous knowledge of LUS can acquire the basic skills following a short intensive training period [24–26].

Up to date, LUS has to be considered as a rapidly expanding field and its use in pediatric care has to be implemented, in order to minimize the use of ionizing radiation and the risk of cancer.

At present, LUS is not included in the management of bronchiolitis but if our results are further upholded in larger, multicentric studies, the use of LUS could be routinely recommended in infants with clinical signs and symptoms of suspected bronchiolitis.

## Conclusions

The diagnosis of bronchiolitis is mainly based on clinical signs and symptoms but lung ultrasound (LUS), given the absence of radiation, may offer a non-invasive, rapid, reproducible, and relatively inexpensive diagnostic tool that could be of exceptional help in the clinical management of bronchiolitis. Thinner chest walls and smaller lung mass make infants and neonates ideal candidates for ultrasound scans, without exposing them to the greater cancer risk of ionizing radiation relative to adults [27, 28].

In pediatric patients with bronchiolitis, LUS reflects the clinical respiratory status and can be used as a rapid and reproducible screening technique to help the physician in the unequivocal identification of infants in need of hospitalization and oxygen supplementation.

In-patient health care costs of bronchiolitis are high [29, 30]. Despite clinical assessment continues to be the gold standard for diagnosis of bronchiolitis, use of LUS for safely reducing hospitalization, might have a great impact on socioeconomic aspect of this disease thereby decreasing health care costs.

In summary, this pilot study demonstrates that the use of LUS in bronchiolitis can be considered as an extension of the clinical evaluation and could be incorporated into clinical algorithms to aid decision-making. Our promising data needs to be confirmed in larger cohort studies also involving critical patients.

## Abbreviations

LUS: Lung ultrasound
SRV: Syncytial respiratory virus
